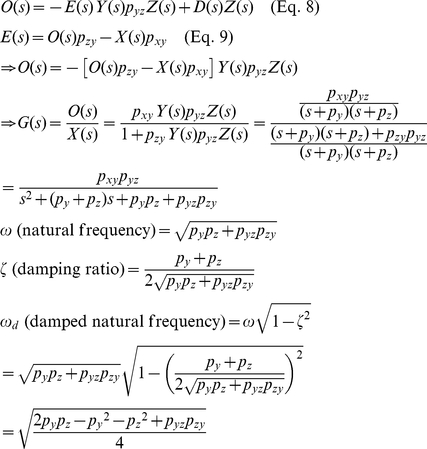# Frequency Domain Analysis Reveals External Periodic Fluctuations Can Generate Sustained p53 Oscillation

**DOI:** 10.1371/journal.pone.0022852

**Published:** 2011-07-28

**Authors:** Yong-Jun Shin, Brandon Hencey, Steven M. Lipkin, Xiling Shen

**Affiliations:** 1 Electrical and Computer Engineering, Cornell University, Ithaca, New York, United States of America; 2 Mechanical and Aerospace Engineering, Cornell University, Ithaca, New York, United States of America; 3 Department of Medicine, Weill Cornell College of Medicine, New York, New York, United States of America; Tulane University Health Sciences Center, United States of America

## Abstract

p53 is a well-known tumor suppressor protein that regulates many pathways, such as ones involved in cell cycle and apoptosis. The p53 levels are known to oscillate without damping after DNA damage, which has been a focus of many recent studies. A negative feedback loop involving p53 and MDM2 has been reported to be responsible for this oscillatory behavior, but questions remain as how the dynamics of this loop alter in order to initiate and maintain the sustained or undamped p53 oscillation. Our frequency domain analysis suggests that the sustained p53 oscillation is not completely dictated by the negative feedback loop; instead, it is likely to be also modulated by periodic DNA repair-related fluctuations that are triggered by DNA damage. According to our analysis, the p53-MDM2 feedback mechanism exhibits adaptability in different cellular contexts. It normally filters noise and fluctuations exerted on p53, but upon DNA damage, it stops performing the filtering function so that DNA repair-related oscillatory signals can modulate the p53 oscillation. Furthermore, it is shown that the p53-MDM2 feedback loop increases its damping ratio allowing p53 to oscillate at a frequency more synchronized with the other cellular efforts to repair the damaged DNA, while suppressing its inherent oscillation-generating capability. Our analysis suggests that the overexpression of MDM2, observed in many types of cancer, can disrupt the operation of this adaptive mechanism by making it less responsive to the modulating signals after DNA damage occurs.

## Introduction

Gene networks exhibit complex dynamic behaviors. Computational and experimental findings suggest that gene networks often contain a small set of recurring motifs [1]. These motifs have unique dynamic properties and perform specific functions. Furthermore, they can display different characteristics under different cellular conditions. If we manage to understand the operations of the motifs in different cellular contexts, we stand a better chance to understand the complex system behavior.

An example of simple motif is the negative feedback loop, a commonly found regulatory mechanism with dual capability of 1) reducing the effects of external noise/fluctuations and 2) generating an oscillatory behavior. For example, negative autoregulation, in which a transcription factor represses the transcription of its own gene, is known to be involved in reducing the effects of noise exerted on the transcription process [2]–[4]. A negative feedback loop can also exhibit oscillatory behavior, which happens during development, immune response, and DNA repair (reviewed in [5]).

**Figure 1:**
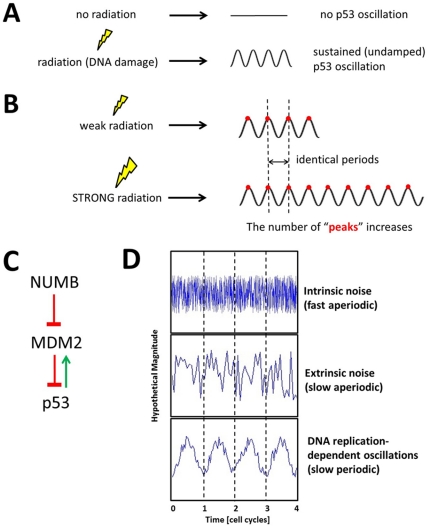
The p53 oscillation. (A) When a cell is exposed to radiation, the p53 levels oscillate in a sustained (undamped) way. (B) A schematic illustration showing that as the extent of DNA damage increases (increased radiation dose) the average number of oscillations (the number of peaks in the figure) also increases but the average duration (period) of each oscillation remains nearly constant. (C) The p53-MDM2 feedback loop. p53 activates MDM2 while MDM2 suppresses p53. NUMB activates p53 by suppressing MDM2. (D) Three types of fluctuation: intrinsic noise or fast aperiodic fluctuations (top), extrinsic noise or slow aperiodic fluctuations (middle), and periodic DNA replication-dependent fluctuations (bottom).

A particularly interesting case is the p53-MDM2 negative feedback loop. The tumor suppressor p53 is one of the most studied proteins in cancer research [6], [7]. Because cells are constantly damaged by various environmental and intrinsic factors, p53 is known to play a key role in deciding whether to repair the damage or activate apoptosis (programmed cell death). In cellular stress conditions, such as radiation-induced DNA damage, the p53 levels are reported to oscillate in a sustained (undamped) way as the p53 suppression by MDM2 is decreased (**Figure 1A**) [8]. One interesting observation is that as the extent of DNA damage increases (due to increased radiation dose), the average number of oscillations also increases but the average duration (period) of each oscillation remains nearly constant (6–7 hours, schematically shown in **Figure 1B**) [9]. The oscillation is thought to be caused by a negative feedback loop formed between p53 and the E3 ubiquitin ligase MDM2 (its human homolog is also known as HDM2) since a negative feedback loop has a capability of generating an oscillation as discussed earlier. In this loop, p53 transcriptionally activates MDM2, while MDM2 degrades p53 via ubiquitination, a process by which proteins are marked for proteasome degradation [10]. Colaluca *et al.* reported a previously unknown function for human NUMB as a regulator of p53. NUMB enters in a tricomplex with p53 and MDM2, thereby preventing ubiquitination and degradation of p53 and increasing the p53 protein levels and activity (**Figure 1C**) [11]. In this paper, NUMB is used to represent a set of cellular factors that can influence the p53-MDM2 feedback loop.

Understanding the stochastic nature of gene networks has been focus of many studies [12], [13] In our study, we want to understand how the p53-MDM2 loop operates under the influence of fluctuations in both normal and stress conditions. The dynamics of gene networks are constantly influenced by various fluctuations (reviewed in [12], [13]). Fluctuations in gene network dynamics originate from variations in transcription, translation, and environmental conditions. There are at least three types of fluctuation that affect gene network dynamics: 1) intrinsic noise or fast aperiodic fluctuations, 2) extrinsic noise or slow aperiodic fluctuations, and 3) periodic DNA replication-dependent oscillations (**Figure 1D**) [14]. Intrinsic noise is caused by the randomness inherent in transcription and translation, key processes for gene expression. Intrinsic noise has been used to analyze gene regulatory links as intrinsic noise can propagate from upstream genes to downstream genes in a path-dependent way [15]. Extrinsic noise arises from the factors that universally affect the expression of all genes in a given cell, such as variations in the number of RNA polymerase, ribosome, etc [12], [13]. The third type of fluctuation is a periodic DNA replication-dependent oscillation, which has been measured in growing and dividing cells [14]. This type fluctuation is also related to DNA repair process. For example, after radiation-induced DNA damage, the stressed cells tend to experience greater fluctuations during the ensuing DNA damage repair process. Because DNA repair utilizes mechanisms that are also used by DNA replication in normal growth conditions, it is likely that the periodic fluctuations displayed by the stressed cells are mechanistically related to periodic DNA replication-dependent oscillation. In this context, we will call the third type of fluctuation as “periodic DNA repair-related fluctuations”, to distinguish it from DNA replication-dependent oscillations.

**Figure 2:**
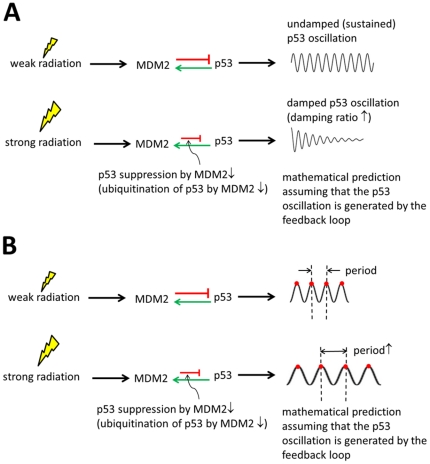
Two contradictions when assuming the p53 oscillation is generated by the feedback loop. (A) Our mathematical analysis illustrates that the damping ratio of the p53 oscillation increases (more damped or less oscillatory) as the radiation dose is stronger. This indicates that when there is more DNA damage there will be less p53 oscillation, which is not consistent with the experimental data shown in **Figure 1B**. (B) It is also mathematically shown that the p53 oscillation period increases as the radiation dose is increased, in contrast to the experimental data that shows it is constant regardless of the radiation dose (**Figure 1B**).

The p53 levels are known to oscillate in cells with radiation-induced DNA damage due to the p53-MDM2 negative feedback loop, which can generate an oscillation, as described earlier. However, recent analysis from Geva-Zatorsky *et al.* suggested that the p53-MDM2 feedback loop can generate only damped oscillations and cannot sustain oscillation without the presence of noise [16]. The incapability of generating a sustained oscillation by a negative feedback loop has also been demonstrated by recent synthetic biology studies [17], [18]. They showed that a negative feedback loop requires a positive autoregulation component, which is missing in the p53-MDM2 loop, in order to generate a sustained oscillation. Furthermore, as discussed in detail later, we found that our mathematical analysis results become contradictory to the experimental evidences schematically shown in **Figure 1B** in case we assume that the p53 oscillation is completely dictated by the p53-MDM2 feedback loop. First, our analysis illustrates that the damping ratio of the p53 oscillation increases (more damped) as the radiation dose is increased (**Figure 2A**). This means that when there is more DNA damage there will be less p53 oscillation, which is not consistent with the experimental evidences shown in **Figure 1B**. Second, the p53 oscillation period can change as the radiation dose varies (**Figure 2B**), in contrast to the experimental result that it is constant regardless of the radiation dose (**Figure 1B**).

**Figure 3:**
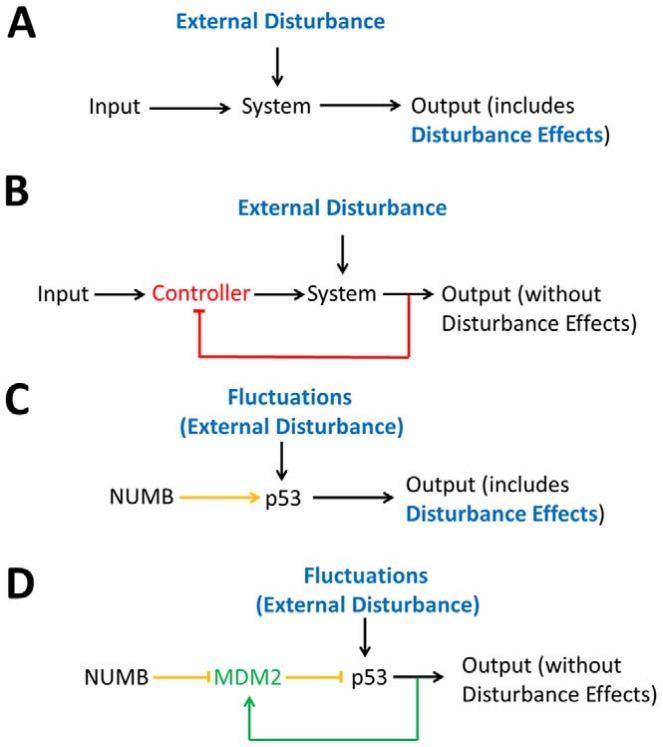
Disturbance rejection (fluctuation filtering). (A) A system receives the input signal in the presence of an external disturbance (shown in blue). As a result, the system generates the output affected by both the input and external disturbance. (B) The external disturbance effects can be filtered (removed) by adding a negative feedback loop and a controller (shown in red) to the system. (C) It is shown that NUMB (input) activates p53 (system) in the presence of fluctuations exerted on p53. (D) MDM2 (controller) and a feedback loop are added to the NUMB-p53 network shown in (C). As a result, the NUMB signal (input) transmitted to p53 (system) is reflected on the output without being affected by fluctuations exerted on p53.

To find out possible explanations for these contradictions, we examined the other feature of the p53-MDM2 feedback loop, the noise/fluctuations filtering, as described earlier. When designed properly, a negative feedback loop can mitigate the impact of unwanted inputs (such as disturbances and fluctuations) that can influence the output of a system [19]. When a system receives desired input signal in the presence of unwanted input or external disturbance (**Figure 3A**, shown in blue), the output is affected by both inputs. The effect of the external disturbance on the output can be minimized by adding a negative feedback loop and a controller (shown in red) to the system (**Figure 3B**). The same mechanism can be used to explain the effects of the p53-MDM2 feedback loop on the p53 level, which is important for the cell. When factors like NUMB tries to regulate p53 activity to perform regular functions, the ever-present fluctuations in the cell can also affect the p53 level (**Figure 3C**). How can a cell remove the effects of these fluctuations exerted on p53? Based on the same strategy described in **Figure 3A and 3B**, MDM2 (shown in green), which acts as a controller and forms a negative feedback loop (also shown in green) with p53, can be inserted between p53 and NUMB (**Figure 3D**). Note that the sign of the feedback loop is positive (shown in green) and the p53 activation by NUMB is represented by two suppressions in series (shown in yellow) in the figure. Since we have MDM2 suppressing p53 and p53 activating MDM2, we end up having a negative feedback control mechanism similar to the one shown in **Figure 3B**. Mathematically, **Figure 3B and 3D** have similar dynamic characteristics as we demonstrate later. Our frequency domain analysis suggests that fluctuations resulting from DNA damage can play a role in modulating the p53 oscillation when the feedback loop stops filtering the fluctuations. This indicates that the p53 oscillation is not completely dependent on the p53-MDM2 negative feedback; instead, the loop has to rely on outside signals to maintain oscillation. In this work, our analysis further shows that, instead of promoting oscillation by decreasing the damping ratio, the damping ratio is increased and the loop stops filtering the fluctuations during DNA damage repair, so that the loop can be more responsive to periodic (oscillatory) DNA repair-related signal described earlier. This is likely a mechanism for cells to synchronize the p53 oscillation with the rest of the repair process in order to achieve the maximum repairing effect.

Most of the above conclusions are derived from our frequency domain analysis of the p53-MDM2 feedback loop. Frequency domain analysis is a commonly used tool in science and engineering. It has been used to analyze the stochastic feature of gene networks [3], [18] and the predictions from a previous frequency domain analysis of the p53 oscillation have been shown to match the experimental measurements [16]. Frequency domain analysis does have its limitations – it can only be performed on a linear model. Nevertheless, frequency analysis has also been widely used for analyzing electrical oscillators which are inherently non-linear devices as well. Because so far no significant non-linear effects have been reported from the studies on p53, we believe frequency domain analysis based on linear approximations will still provide insights into the operation of this feedback motif.

## Results

### Simple gene regulation is a low-pass filter

Before moving into complex feedback network configurations, such as the p53-MDM2 feedback loop, it is useful to examine the stochastic characteristics of simple gene regulation in frequency domain. Simple gene regulation is a two-gene network that serves as a basic building block for constructing more complex networks. In this section, we present a simple gene regulation example from *E. coli*, as there are experimental data available regarding the three different types of fluctuations described in the previous section [14]. However, as the filtering function of a negative feedback loop is a generic mechanism that can be universally observed in both prokaryotes and eukaryotes, it is assumed that the conclusion derived from our prokaryotic example can be applied to the eukaryotic case we are interested in. An ordinary differential equation (ODE) for simple gene regulation (gene *x* activates gene *y*) can be expressed as
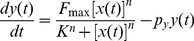
(1)where *x*(*t*) and *y*(*t*) stand for the concentrations of protein *x* and *y* as a function of time *t*. *F_max_* is the maximal level of the *y* protein production (in units of concentration per unit time) that is reached when *x*(*t*)≫*K*. *K* is the concentration of *x*(*t*) at which half-maximal production of *y* protein is reached and *n* is the Hill coefficient. *P_y_* is a degradation/dilution parameter that affects the rate at which *y* decreases [20]. Eq. 1 can be transformed into a linearized form as
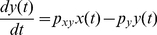
(2)where *p_xy_* is a parameter that determines the effect of *x* protein on the production of *y* protein. As described in the previous section, this linearization step is critical for frequency domain analysis. More detailed information about our linearization can be found in one of our previous works [18].

**Figure 4:**
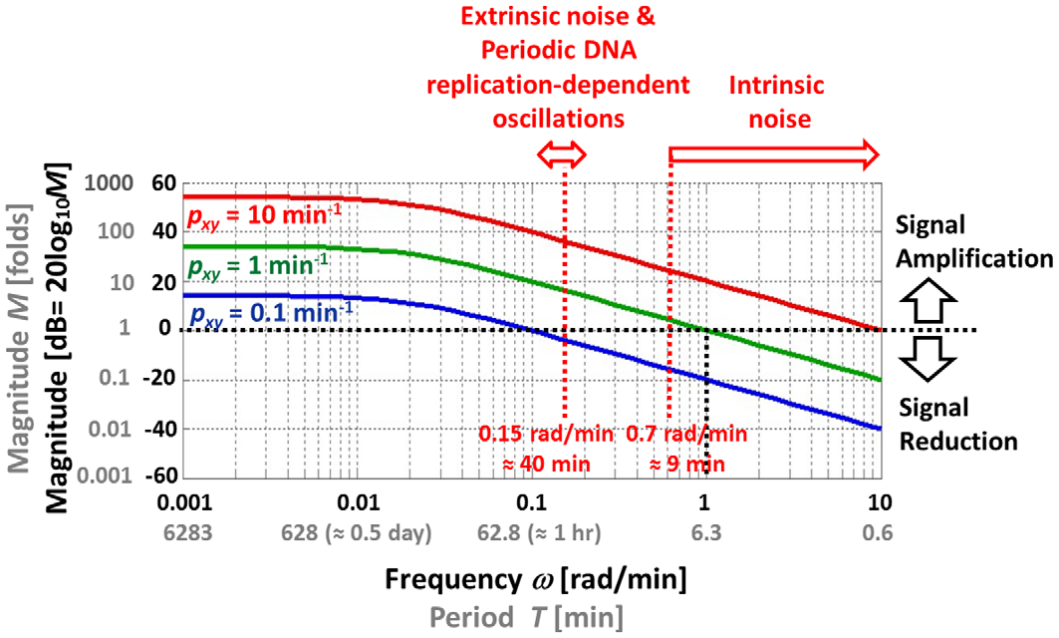
Bode plot of simple gene regulation. There are three plots with *p_y_* = 0.02 min^−1^ and *p_xy_* = 10 (red), 1 (green), and 0.1 (blue) min^−1^. On the horizontal axis, [rad/min] is used for the unit of angular frequency *ω* and [min] is used for the unit of equivalent period *T* (*T* = 2*π*/*ω*). The vertical axis represents the magnitude by which the system amplifies or reduces the input. The magnitude is shown in both folds (*M*) and dB (20log_10_ *M*). The figure illustrates that simple gene regulation can either amplify or reduce intrinsic noise, extrinsic noise, and periodic DNA replication-dependent oscillations depending on the value of *p_xy_*.

Using the Laplace transform, the transfer function *G*(*s*) that relates the input *x*(*t*) to the output *y*(*t*) in frequency domain can be shown as (see **Methods**) [18]

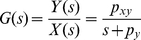
(3)where *X*(*s*) and *Y*(*s*) are the Laplace transform of *x*(*t*) and *y*(*t*). The transfer function *G*(*s*) represents a system that receives the input *X*(*s*) and produces the output *Y*(*s*) after processing the input. Gene expression is a slow process that takes minutes to hours. For stable proteins in bacteria, the response time (*T*_1/2_), the time for *y* to reach one half of the steady state after activated by *x*, is approximately 30 min and *p_y_* can be approximated as (see **Methods**) [20]

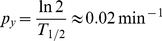
(4)Frequency response of a system is the measure of the system's response to the input in frequency domain and can be illustrated graphically using a Bode plot [19]. **Figure 4** shows the Bode plot (magnitude plot) of simple gene regulation with *p_y_* = 0.02 min^−1^ and different *p_xy_* values (0.1, 1, and 10 min^−1^). On the horizontal axis, [rad/min] is used for the unit of angular frequency *ω* and [min] is used for the unit of equivalent period *T* (*T* = 2*π*/*ω*). The vertical axis represents the magnitude by which the system amplifies or reduces the input. The magnitude is shown in both folds (*M*) and dB (20log_10_ *M*). For example, in the case of *p_xy_* = 1 min^−1^ (green plot in **Figure 4**), when the frequency *ω* of the input signal is approximately 1 rad/min (or the period is about 6.3 min as shown in the figure), the magnitude *M* is 1 fold (or 0 dB) meaning that the input is neither amplified nor reduced. This can be mathematically verified as following. When *M* is 1 fold, the mathematical relation between *ω* and *p_y_* can be described as (see **Methods**)
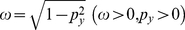
(5)In Eq. 5, when *p_y_* is 0.02 min^−1^ *p_y_* ^2^ becomes very small (0.0004 min^−2^) driving *ω* close to 1 rad/min, as approximated earlier from **Figure 4**. Even though the *p_xy_* value is likely to vary from cell to cell, the Bode plots in **Figure 4** illustrate that simple gene regulation is a low-pass filter that amplifies low-frequency components (low *ω*) and reduces high-frequency components (high *ω*). Note these amplification and reduction are not absolute but relative terms that depend on the parameter values.

**Figure 5:**
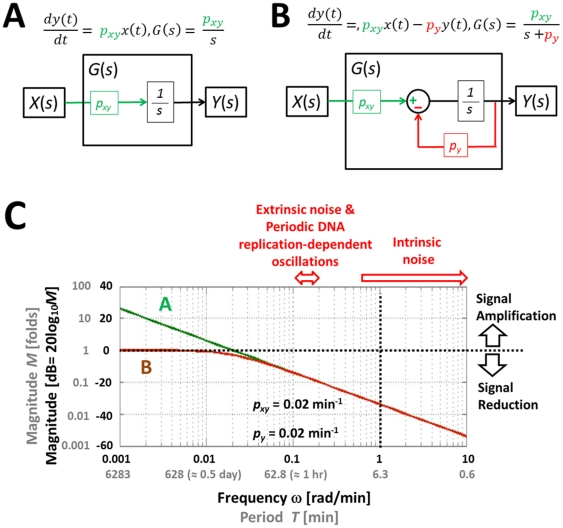
Simple gene regulation with and without the degradation/dilution term of **Eq. 1**. (A) The ODE, transfer function, and block diagram of simple gene regulation in which the second or degradation/dilution term is removed. (B) The ODE (equivalent to Eq. 1), transfer function, and block diagram of simple gene regulation with the second term intact. Note that the block diagram has a negative feedback component, which could be hardly revealed by examining Eq. 2. (C) The Bode plot for (A) and (B). The figure illustrates that low-frequency signals are increasingly amplified as their frequencies decrease in the case of (A). On the other hand, (B) exhibits a “plateau-like” curve (brown), indicating that the amplifying magnitude remains constant regardless of the frequency decrease. Note that their capabilities to filter intrinsic noise and other types of fluctuation are quite similar.

The time scale (or the period *T*) is one of the differences between intrinsic noise and other types of fluctuations (extrinsic noise and periodic DNA replication-dependent oscillations) [14]. Time-lapse microscopy showed that the time scale for intrinsic noise was less than 9 min (or *ω*>0.7 rad/min) whereas the time scale for extrinsic noise and periodic DNA replication-dependent oscillations was about 40 min (*ω*≈0.15 rad/min) in bacteria (**Figure 4**) [14]. Depending on the value of *p_xy_*, simple gene regulation can either amplify or reduce intrinsic noise, extrinsic noise, and periodic DNA replication-dependent oscillations (**Figure 4**). For example, when *p_xy_* = 10 min^−1^ (red plot in the figure), all three types of fluctuations are amplified. On the contrary, when *p_xy_* = 0.1 min^−1^(blue plot in the figure), most of them are reduced. We have an interesting case when *p_xy_* = 1 min^−1^ (green plot in the figure). In this case, extrinsic noise and periodic DNA replication-dependent oscillations are amplified whereas intrinsic noise is mostly reduced. The role of the degradation/dilution rate of *y*(*t*) can also be illustrated using a Bode plot. By forming a negative feedback (**Figure 5B**), the degradation/dilution term enables the transfer function to have a flat response at low frequencies while attenuating signals at high frequencies (**Figure 5C**). On the other hand, without the degradation term, the open-loop response amplifies low-frequency signals (**Figure 5A and 5C**, also see **Methods** for the transfer function derivation).

### Effects of autoregulation on noise reduction

In the previous section, we showed that simple gene regulation can selectively amplify or filter different types of fluctuations by varying the key parameter *p_xy_*. The problem of simple gene regulation is that when regulatory input signals (wanted input) are mixed with those fluctuations (unwanted input) it cannot selectively filter the unwanted input only. This section examines if negative autoregulation, known to be involved in reducing the effects of noise or fluctuations [2]–[4], has such capability of selective filtering.

**Figure 6:**
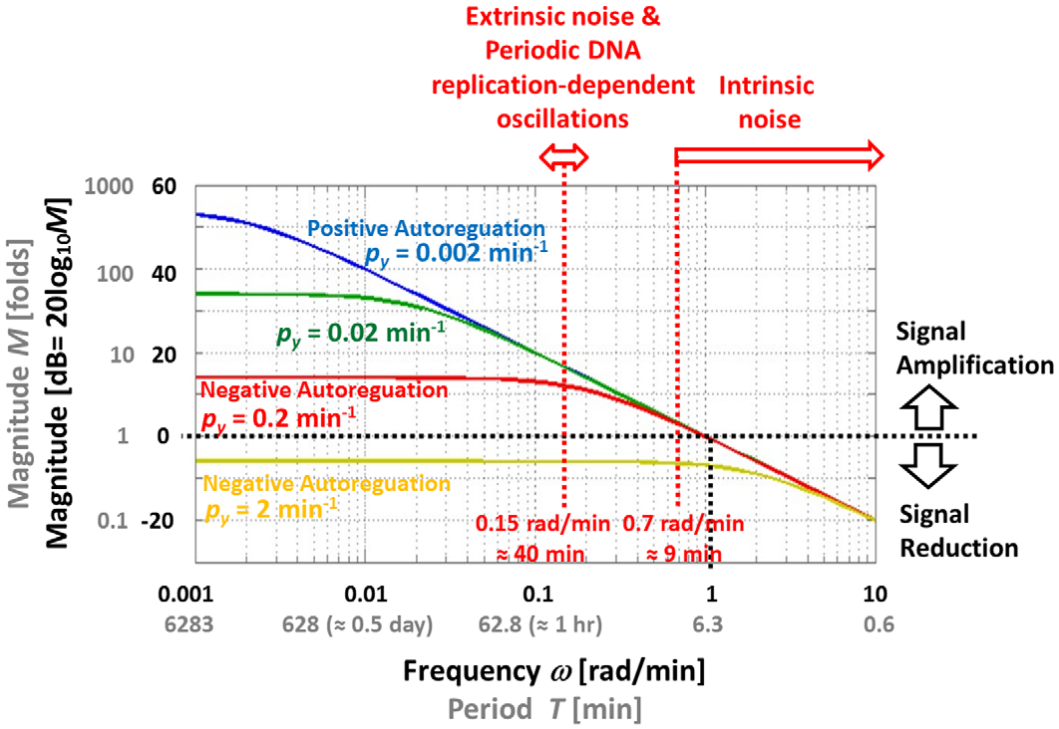
Bode plot of autoregulations. There are four plots with *p_xy_* = 1 min^−1^ and *p_y_* = 0.002 (blue), 0.02 (green), 0.2 (red), and 2 (yellow) min^−1^. On the horizontal axis, [rad/min] is used for the unit of angular frequency *ω* and [min] is used for the unit of equivalent period *T* (*T* = 2*π*/*ω*). The vertical axis represents the magnitude by which the system amplifies or reduces the input. The magnitude is shown in both folds (*M*) and dB (20log_10_ *M*). The figure shows that positive autoregulation (blue) similarly amplifies extrinsic noise and periodic DNA replication-dependent oscillations compared to simple gene regulation (green), indicating that it cannot play a role in filtering the effects of such fluctuations. Negative autoregulation clearly decreases the magnitude of amplification of those fluctuations (red) or even reduces it (yellow). However, it is also shown that entire low-frequency signals are affected in a non-selective way.

Autoregulation is a form of feedback loop that consists of one simple gene regulation and one feedback loop. Negative autoregulation occurs when a protein represses the transcription of its own gene (negative feedback) and positive autoregulation occurs when a protein enhances its own protein production rate [20]. Assuming *p_xy_* = 1 min^−1^, the effects of adding autoregulatory feedback loops to simple gene regulation on the transfer function *G*(*s*) shown in Eq. 3 can be expressed as (see **Methods**) [18]

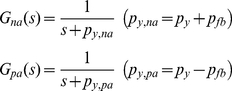
(6)where *na*, *pa*, and *fb* stand for negative autoregulation, positive autoregulation, and feedback, respectively. *p_fb_* represents the strength of a feedback loop. Note that negative autoregulation increases the value of *p_y_* by *p_fb_*, while positive autoregulation decreases *p_y_* by *p_fb_*. The effects of autoregulations on simple gene regulation in frequency domain are shown in **Figure 6**. The *p_y_* value is fixed at 0.02 min^−1^ as discussed in Eq. 4. By having *p_fb_* as 0.18 and 1.98 rad/min, 0.2 and 2 rad/min were used as the *p_y,na_* values in the case of negative autoregulation (red and yellow plots). For positive autoregulation, *p_y,pa_* was 0.002 rad/min with *p_fb_* = 0.018 (blue plot). Our plot shows that positive autoregulation amplifies extrinsic noise and periodic DNA replication-dependent oscillations in a similar fashion to simple gene regulation (green plot). On the contrary, negative autoregulation clearly decreases the amplification magnitude of those slowly-varying fluctuations (red plot) or even reduces it (yellow plot). However, it does not distinguish regulatory input signals (wanted input) from noise or fluctuations (unwanted input) as long as their frequencies overlap, meaning that it does not have the capability of desired selective filtering. This suggests that neither positive nor negative autoregulation is a satisfactory mechanism to filter out unwanted fluctuations while preserving regulatory signals for p53.

### The p53-MDM2 feedback loop is an input-specific filter that can selectively remove the effects of unwanted fluctuations

**Figure 7:**
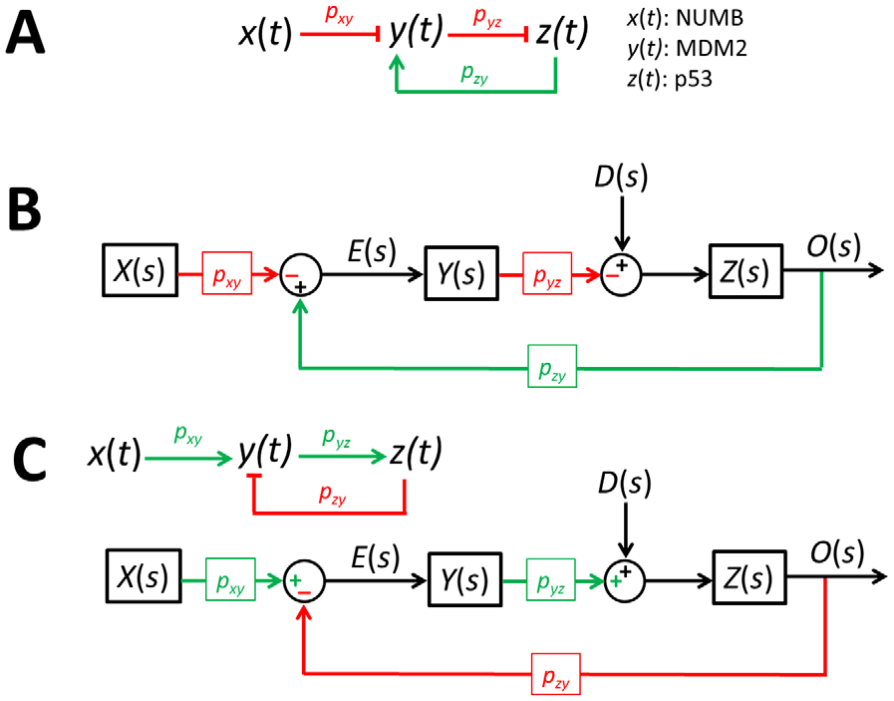
p53 network. (A) Schematic drawing of the p53-network. (B) Block diagram representation. *X*(*s*), *Y*(*s*), and *Z*(*s*) are the Laplace transform of *x*(*t*), *y*(*t*), and *z*(*t*), respectively. *O*(*s*) is the Laplace transform of the output and *E*(*s*) is the transform of an error *e*(*t*), which is the difference between the input and output. *D*(*s*) is a disturbance representing fluctuations exerted on p53. (C) A network configuration discussed in **Figure 3B**. It has an equivalent oscillation filtering effect as the configuration shown in (B).

A mathematical model for our p53-MDM2 feedback loop can be expressed as
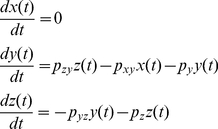
(7)where x(*t*), y(*t*), and z(*t*) stand for NUMB, MDM2, and p53, respectively (**Figure 7A**). *p_xy_*, *p_yz_*, *p_zy_*, *p_y_*, and *p_z_* are the parameters. Using the Laplace transform, Eq. 7 can be represented as a block diagram as shown in **Figure 7B** [18]. *X*(*s*), *Y*(*s*), and *Z*(*s*) are the Laplace transform of *x*(*t*), *y*(*t*), and *z*(*t*), respectively. *O*(*s*) is the Laplace transform of the output and *E*(*s*) is the transform of an error *e*(*t*), which is the difference between the input and output. *D*(*s*) is a disturbance representing unwanted fluctuations exerted on p53.

From **Figure 7B**, the Laplace transform of the output *O*(*s*) can be expressed as [19]

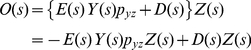
(8)Since *E*(*s*) is the difference between the input and output
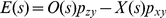
(9)Substituting Eq. 9 into Eq. 8 and solving for *E*(*s*), we get (see **Methods**) [19]

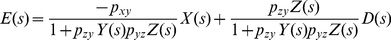
(10)The second term in Eq. 10 can be regarded as a component of *E*(*s*) that is contributed by *D*(*s*). Denoting its corresponding time domain function as *e_D_*(*t*), the *E_D_*(*s*) or Laplace transform of *e_D_*(*t*) can be expressed as
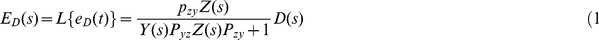
(11)Using the final value theorem, we can show the steady-state error due to the disturbance as [19]

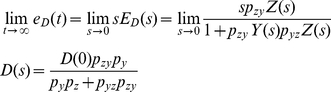
(12)where *Y*(*s*) = 1/(*s*+*p_y_*) and *Z*(*s*) = 1/(*s*+*p*_z_). Eq. 12 gives us an insight that the steady-state error or the error caused by the fluctuations exerted on p53 can be minimized by increasing *p_yz_*, the strength by which *y* (MDM2) suppresses *z* (p53), or the rate at which MDM2 ubiquitinates p53. The equation also illustrates that the p53-MDM2 feedback loop can filter out the fluctuations while not influencing the NUMB signal so that it can be transmitted to p53 without being influenced by the fluctuations. Following the identical mathematical derivation steps used for Eq. 7, it can be shown that two different network configurations illustrated in **Figure 7B and 7C** have the same effect (removal of the fluctuations exerted on p53), even though their activation and suppression signs are reversed. This point was also mentioned earlier in **Figure 3**.

**Figure 8:**
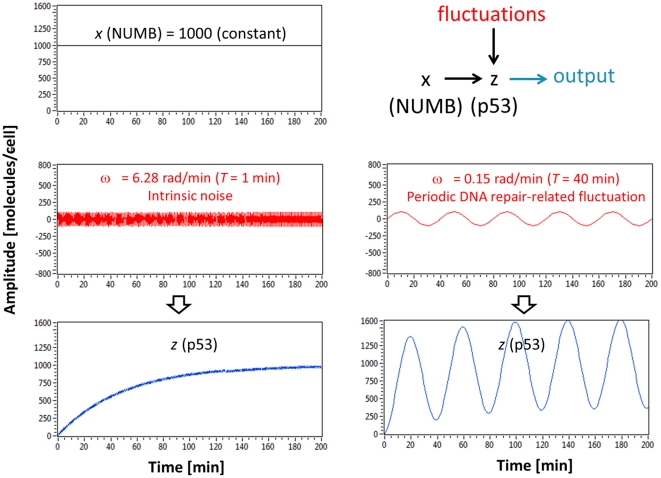
Simple gene regulation cannot filter periodic DNA repair-related fluctuation. A simulation result shows that simple gene regulation (*x* activating *z* or NUMB activating p53) can reduce intrinsic noise but amplify periodic DNA repair-related fluctuation. At top left, *x* (NUMB) is assumed to be constant (1000 molecules/cell). On the left of the figure, it is shown that intrinsic noise (period *T* = 1 min and amplitude = 100 molecules/cell) is filtered by simple gene regulation. However, periodic DNA repair-related fluctuation (shown on the right, period longer period *T* = 40 min) with the same amplitude (100 molecules/cell) is not reduced but amplified. In order to explicitly illustrate the frequency-dependence, sinusoidal waveforms are used as fluctuation signals.

**Figure 9:**
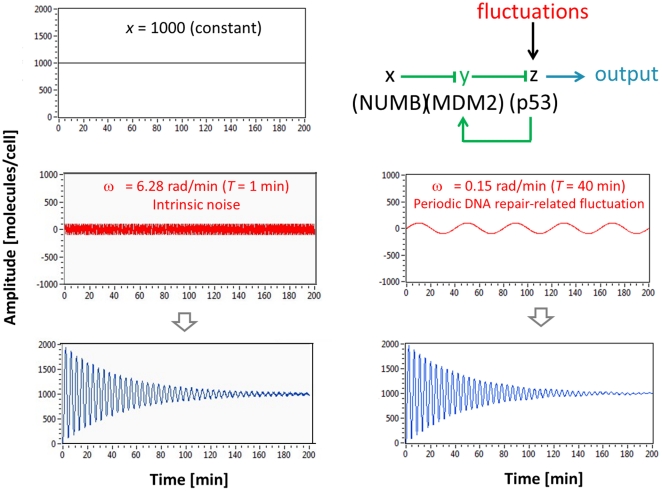
p53-MDM2 feedback loop can filter DNA repair-related oscillatory signals. At top left, *x* is assumed to be constant ( = 1000 molecules/cell). On the left of the figure, it is shown that intrinsic noise (period *T* = 1 min and amplitude = 100 molecules/cell) is filtered by the feedback loop. Periodic DNA repair-related fluctuation (period *T* = 40 min) with the same amplitude (100 molecules/cell) is also filtered as shown on the right.

Using simulation, we can compare the fluctuation filtering capabilities of simple gene regulation and negative feedback loop. A case of simple gene regulation, where *x* (NUMB) activates *z* (p53), is shown in **Figure 8**. At top left, it is shown that *x* is constantly expressed (1000 molecules/cell). High-frequency fluctuation (period *T* = 1 min and amplitude = 100 molecules/cell), which represents intrinsic noise, is shown at middle left. Since we are especially interested in the effects of “periodic DNA repair-related fluctuations”, fluctuations with longer (slower) period *T* = 40 min and the same amplitude (100 molecules/cell) are shown at middle right. In order to explicitly illustrate the frequency-dependence, sinusoidal waveforms are used as fluctuation signals. The total input is the sum of the constant NUMB value (1000 molecules/cell) and either intrinsic noise or periodic DNA repair-related fluctuation values. Our simple gene regulation example can reduce or filter intrinsic noise but amplify periodic DNA repair-related fluctuation (bottom in **Figure 8**), as predicted previously in **Figure 4** (green plot). When a feedback loop and controller *y* (MDM2) are added to the simple gene regulation case, which cannot filter periodic DNA repair-related fluctuation exerted on *z* (p53), the modified circuit can eventually filter both intrinsic noise and periodic DNA repair-related fluctuation, driving the steady-state error (the difference between the NUMB signal and the output) to a minimum (**Figure 9**). This illustrates that the p53-MDM2 feedback loop is an input-specific filter that allows the regulatory input signal (NUMB) to pass but selectively removes unwanted input signals (fluctuations).

**Figure 10:**
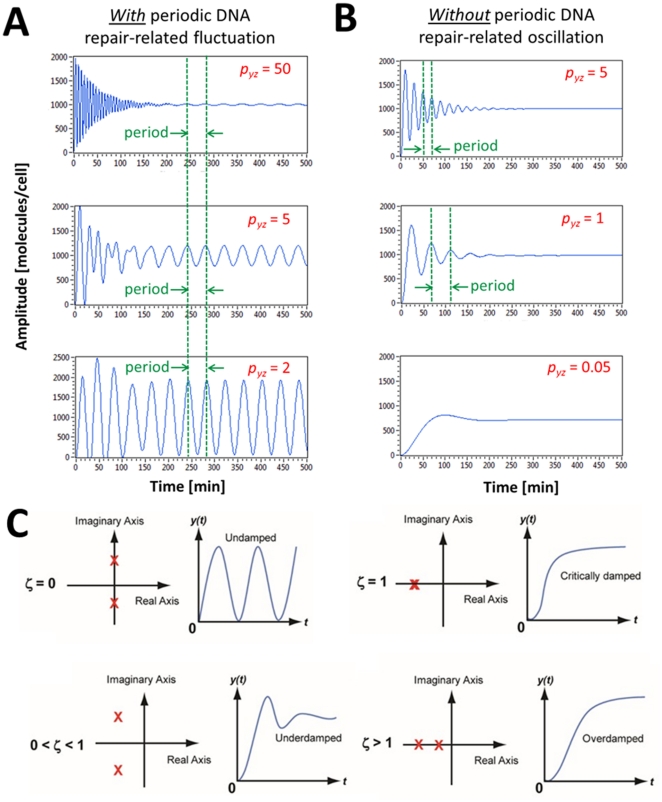
The effects of the *p_yz_* strength. (A) As *p_yz_* is decreased, periodic DNA repair-related fluctuation (*T* = 40 min and amplitude = 100 molecules/cell) is filtered less, so the p53 levels fluctuate more. Note that the period of the oscillation does not change as the *p_yz_* value is decreased. (B) Without periodic DNA repair-related fluctuation, the decrease in *p_yz_* will also decrease the p53 oscillatory behavior (more damping). The period of the oscillation increases as the *p_yz_* value is decreased. (C) Step responses of a second-order system with respect to the damping ratio *ζ* (the poles of the transfer function are shown as X on the complex plane) [19].

It is important to examine the role of *p_yz_*, the strength by which *y* (MDM2) suppresses *z* (p53), in detail since it is experimentally known that p53 oscillates as *p_yz_* is decreased (or the p53 ubiquitination by MDM2 is decreased) upon DNA damage [8]. In the previous section, Eq. 12 gave us an insight that the fluctuations exerted on p53 can be minimized or filtered more by increasing *p_yz_*, meaning that the fluctuations are “filtered less” when *p_yz_* decreases. Our mathematical insight and the previous experimental observation indicate that DNA damage causes the *p_yz_* decrease (less p53 ubiquitination by MDM2), which results in less filtering of periodic fluctuation affecting p53 and thereby more p53 level fluctuation. This is well illustrated in **Figure 10A**. As *p_yz_* is decreased, the periodic DNA repair-related fluctuation (*T* = 40 min and amplitude = 100 molecules/cell) exerted on p53 is filtered less and the p53 level fluctuates more. Our finding illustrates that the p53 oscillation can be generated by periodic DNA repair-related fluctuation.

### The damping ratio of the p53-MDM2 feedback loop is increased upon DNA damage

The natural oscillating frequency (*ω*), damping ratio (*ζ*), and damped natural frequency (***ω_d_***) of the p53-MDM2 feedback loop shown in **Figure 7A** can be expressed as (see **Methods**) [18]

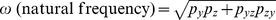
(13)

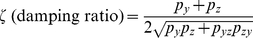
(14)

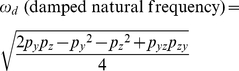
(15)Eq. 14 tells us that if we decrease *p_yz_*, which is in the denominator, the damping ratio *ζ* will increase (more damping) and there will be a decreased oscillating behavior as shown in **Figure 10C**. This mathematical insight is well-illustrated in **Figure 10B**. In the absence of periodic DNA repair-dependent fluctuation, the p53 level exhibits a less oscillatory (more damped) behavior as *p_yz_* decreases. As mentioned earlier, p53 oscillates as the p53 ubiquitination by MDM2 is decreased (or *p_yz_* is decreased) upon DNA damage [8]. Eq. 14 and **Figure 10B** show that this *p_yz_* decrease will increase the damping ratio and make the p53 level less oscillatory, in contrast to the assumption that the feedback loop mechanism contributes to the p53 oscillation, as we discussed in **Figure 2A**. It is also known that the overexpression of MDM2 (or the *p_yz_* increase) is tumorigenic (capable of forming tumors) by blocking the p53 oscillation. Again, Eq. 14 and **Figure 10B** illustrate that the MDM2 overexpression will not decrease but increase the oscillatory behavior due to the feedback loop mechanism by decreasing the damping ratio (or increasing *p_yz_*). On the other hand, **Figure 10A** shows that the MDM2 overexpression (*p_yz_* increase) will decrease the p53 oscillation because DNA repair-related signals that are required to sustain the oscillation will be filtered by the feedback loop. Overall, **Figure 10** suggests that the p53-MDM2 feedback loop cannot completely sustain the p53 oscillation by itself. Instead, there likely exist periodic DNA repair-related fluctuations from other cellular events that modulate and help sustain this oscillatory behavior. By being adaptive, the feedback loop keeps the p53 oscillation in sync with the rest of the DNA repair process in the cell.

### The period of the p53 oscillation is unchanged upon different extents of DNA damage

It was mentioned earlier that when the extent of DNA damage increases, the average duration (period) of each oscillation remains nearly constant even though the average number of p53 oscillations increases (**Figure 1B**) [9]. This experimental observation is consistent with our simulation result shown in **Figure 10A**. In this simulation, different extents of DNA damage (or radiation dose) correspond to different values of *p_yz_*. The period of the p53 oscillation remains the same even though *p_yz_* changes because it is in sync with the constant (*T* = 40 min in this example) period of DNA repair-related oscillatory signal. On the other hand, Eq. 15 illustrates that damped natural frequency (*ω*) (or period *T* = 2π/*ω*) changes as the *p_yz_* value varies when the oscillating behavior is originated from the feedback loop. **Figure 10B** also demonstrates that the damped natural frequency *ω_d_* and period *T* changes as *p_yz_* varies, indicating that the p53 oscillation is not completely determined by the p53-MDM2 feedback loop. In summary, the simulation result supports that the p53 oscillation is modulated by the periodic DNA repair-related fluctuations.

## Discussion

The p53-MDM2 negative feedback loop has dual functions: generating oscillation and filtering disturbances. Using frequency domain analysis, we demonstrated that the p53-MDM2 feedback mechanism adapts to different cellular contexts. It normally filters fluctuations exerted on p53, however, upon DNA damage, it stops filtering noise and fluctuations. The DNA repair-related oscillatory signals can then be passed on to modulate the p53 oscillation. We reasoned that the p53 oscillation may not be completely dictated by the inherent p53-MDM2 feedback mechanism alone based on the following arguments: 1) upon DNA damage, the damping ratio of the feedback loop increases rendering the p53 level less oscillatory, and 2) experimental evidences suggest that the period of the p53 oscillation remains nearly constant when facing different extents of DNA damage [9].

Our frequency domain analysis focused on two parameters, the damping ratio and damped natural frequency. Especially, we examined how *p_yz_* (the strength by which MDM2 suppresses p53) affects those parameters. As described earlier, it has been known that p53 oscillates as *p_yz_* (the p53 ubiquitination by MDM2) is decreased upon DNA damage. However, this raises a question since the *p_yz_* decrease indicates that the feedback loop is “weakened”, which results in an increased damping ratio (or decreased oscillatory behavior). In the extreme case, *p_yz_* is decreased to zero and there should be no oscillatory behavior if the oscillation is solely originated from the feedback loop. However, experiments show that the oscillation in fact increases when there is more DNA damage, which presumably further decreases *p_yz_*. This seeming contradiction can be explained if we take into consideration the loop's normal filtering function before DNA damage. In this regard, the *p_yz_* decrease or “weakened” feedback loop signifies not only less oscillation but also less filtering. The feedback loop no longer actively removes the fluctuations originated outside the feedback loop. These fluctuation signals can play a role in modulating the p53 oscillation and keep the p53 oscillation in sync with periodic DNA repair-related signals.

The overexpression of MDM2 has been observed in many types of cancer (reviewed in [21]). Since p53 is a tumor repressor and MDM2 suppresses p53 through ubiquitination, inhibiting MDM2 activity in tumors has been considered as cancer therapeutics (reviewed in [22]). Our analysis indicates that the overexpression of MDM2, or the increase of *p_yz_*, augments the filtering of the p53-MDM2 feedback loop and makes the loop less responsive to the modulating signals after DNA damage occurs. In other words, as MDM2 is constantly overexpressed, the adaptability of the feedback loop is lost. This loss of adaptability plays into the advantage of cancer cells, because p53 will not be able to respond to the DNA damage to either repair the damage or cause apoptosis. Based on our understanding of the p53-MDM2 dynamics, novel therapeutics should ideally work to restore the lost adaptability of the p53-MDM2 feedback loop.

Our computational analysis suggests that future experiments might want to search for factors that are related to the DNA repair process and display oscillatory behaviors. These factors can potentially serve as the DNA repair-related signals that influence the p53 oscillation. They can then be knocked down in the cells that will be treated with radiation; afterwards the p53 levels can be measured to see whether p53 still oscillates, and if so, whether the frequency is changed. If our hypothesis is correct, p53 will stop oscillating without the input from the periodic DNA-repair related signals.

## Methods

### Derivation of the transfer function G(s) for simple gene regulation (Eq. 3)



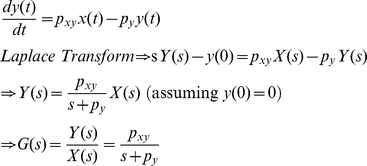



### Calculation of the response time (T_1/2_)



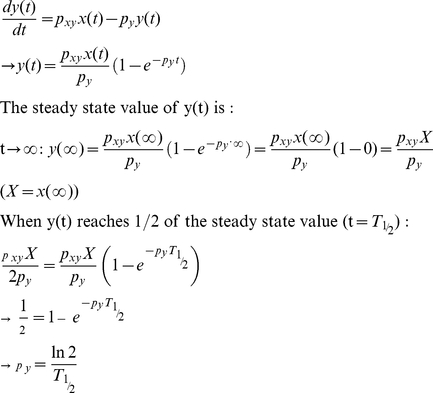
Mathematical relation between *ω* and *p_y_* when *M* is 1 fold (Eq. 5)
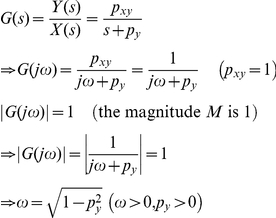



### Derivation of the transfer function G(s) for simple gene regulation without the degradation/dilution term (Figure 5A)



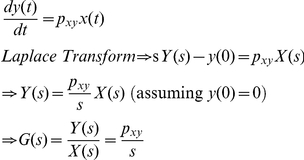



### Transfer function G(s) for autoregulations (Eq. 6) [18]



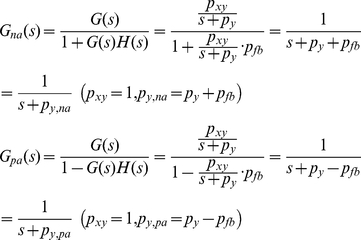



### Derivation of *E*(*s*) (Eq. 10)



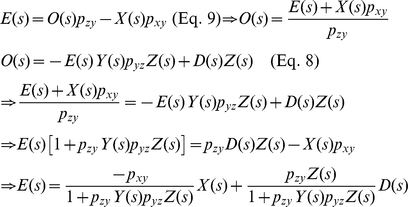



### Derivation of the natural oscillating frequency (*ω*) , the damping ratio (ζ), and damped natural frequency (*ω_d_*) of the p53-MDM2 feedback loop (Eq. 13, 14, and 15)